# Pulsed Electrical Stimulation of the Human Eye Enhances Retinal Vessel Reaction to Flickering Light

**DOI:** 10.3389/fnhum.2019.00371

**Published:** 2019-10-22

**Authors:** Stefanie Freitag, Alexander Hunold, Matthias Klemm, Sascha Klee, Dietmar Link, Edgar Nagel, Jens Haueisen

**Affiliations:** ^1^Institute for Biomedical Engineering and Informatics, Technische Universität Ilmenau, Ilmenau, Germany; ^2^Ophthalmic Private Practice, Rudolstadt, Germany

**Keywords:** pulsed electrical stimulation, transcranial direct current stimulation (tDCS), flicker light stimulation, dynamic vessel analysis, retinal vessel diameter, vasodilation

## Abstract

Recent studies indicate therapeutic benefits of electrical stimulation in cases of specific ophthalmic diseases that are associated with dysfunctional ocular microcirculation. This suggests effects of electrical stimulation on vascular functions. In the present study, we investigated the effects of electrical stimulation on retinal vessel reactions using dynamic vessel analysis (DVA). Eighty healthy subjects were randomly assigned to one of three groups receiving electrical stimulation with different current intensities: 400 μA (*n* = 26); 800 μA (*n* = 27); 1200 μA (*n* = 27). The electrode montage for electrical stimulation consisted of a ring-shaped active electrode surrounding one eye and a square return electrode at the occiput. Rectangular, monophasic, positive current pulses were applied at 10 Hz for a duration of 60 s per stimulation period. DVA was used to observe the stimulation-induced reactions of retinal vessel diameters in response to different provocations. In three DVA measurements, three stimulus conditions were investigated: flicker light stimulation (FLS); electrical stimulation (ES); simultaneous electrical and flicker light stimulation (ES+FLS). Retinal vasodilation caused by these stimuli was compared using paired *t*-test. The subjects receiving electrical stimulation with 800 μA showed significantly increased retinal vasodilation for ES+FLS compared to FLS (*p* < 0.05). No significant differences in retinal vessel reactions were found between ES+FLS and FLS in the 400 and 1200 μA groups. No retinal vasodilation was observed for ES for all investigated current intensities. The results indicate that positive pulsed electrical stimulation of an adequate intensity enhances the flicker light-induced retinal vasodilation.

## Introduction

Electrical stimulation of the eyes has demonstrated positive effects in patients suffering from ophthalmic diseases, such as retinitis pigmentosa (Schatz et al., 2011, 2017), age-related macular degeneration (Anastassiou et al., 2013; Chaikin et al., 2015), retinal artery occlusion (Inomata et al., 2007; Oono et al., 2011; Naycheva et al., 2013), and optic neuropathy (Gall et al., 2011, 2016; Sabel et al., 2011). To evaluate the success of such treatments in humans, common ophthalmic parameters such as visual acuity, visual field, and multifocal electroretinography were evaluated and revealed improvements. Several studies in animal disease models support these findings showing prolonged survival of retinal ganglion cells (RGCs) (Morimoto et al., 2002; Miyake et al., 2007; Tagami et al., 2009; Wang et al., 2011) and photoreceptors (Morimoto et al., 2007, 2012; Ni et al., 2009) after electrical stimulation.

The beneficial therapeutic impacts are associated with an upregulation of neurotrophic factors that are released from Müller cells, including insulin-like growth factor 1 (IGF-1) (Morimoto et al., 2005; Sato et al., 2008b), brain-derived neurotrophic factor (BDNF) (Sato et al., 2008a), and ciliary neurotrophic factor (CNTF) (Ni et al., 2009). The Müller cells are a substantial type of retinal glial cells that assume regulatory functions in the retina to modulate neuronal activity and blood flow by controlling voltage-gated channels, neurotransmitter receptors, and neuroactive substances (Newman and Reichenbach, 1996; Newman, 2015). However, the neuroprotective effects depend on the parameters of the electrical stimulation, such as intensity, duration, and waveform (Morimoto et al., 2010).

The abovementioned ophthalmic diseases are associated with dysfunctions in ocular microcirculation (Pemp and Schmetterer, 2008; Konieczka et al., 2012). Thus, the improvement of visual functions in such cases might also be related to the observed enhancement of chorioretinal blood flow in healthy humans several hours after transcorneal electrical stimulation (Kurimoto et al., 2010). In addition, Mihashi et al. (2011) showed in cats that transcorneal electrical stimulation upregulates the retinal blood flow already within a few seconds after the stimulation by evaluating retinal reflectance changes. However, a detailed investigation of the effects of electrical stimulation on microcirculation in the human eye is still pending, but is very important for the understanding of electrical stimulation-induced mechanisms.

A non-invasive method for the *in vivo* observation of retinal microcirculation in humans is the dynamic vessel analysis (DVA) (Garhofer et al., 2010; Link et al., 2011), which is based on the measurement of diameter changes of retinal vessels under physiological provocation with flickering light. Endothelial function is essential for the regulation of retinal vessel diameters and the associated blood flow changes. We assume that electrical stimulation can affect the endothelial function. Therefore, the present study investigated retinal vessel reactions in response to electrical stimulation using DVA for the first time. We tested the hypothesis that flicker light-induced vasodilation is increased by electrical stimulation of the eye.

## Materials and Methods

The study was approved by the local ethics committee of the Friedrich Schiller University Jena, Germany. All procedures complied with the Declaration of Helsinki and the subjects gave their written informed consent before participating in the study.

### Subjects

Retinal vessel reactions to different stimuli were examined in 80 young, healthy, non-smoking Caucasian subjects (age range: 20–37 years; one eye). They were randomly assigned to one of three age-matched groups (*p* < 0.05) that received electrical stimulation with different current intensities: 400 μA (*n* = 26; 8 male; 18 female; mean age: 25.1 ± 4.0 years); 800 μA (*n* = 27; 12 male; 15 female; mean age: 24.3 ± 1.6 years); 1200 μA (*n* = 27; 13 male; 14 female; mean age: 25.6 ± 3.0 years). None of the subjects suffered from ophthalmic, neurological, vascular, and systemic diseases or received any regular medication (except hormonal contraceptives). Additional exclusion criteria were epilepsy, seizures, head injuries, electronic/metallic implants as well as anatomical anomalies in the head or upper body, smoking, and pregnancy. Before participating in the experiments, all subjects passed an ophthalmic examination (measurement of visual acuity; objective refraction; non-contact tonometry; slit lamp microscopy; ophthalmoscopy) to ensure that there were no clouding of the refractory media and no vision impairments outside the predefined limits for the Retinal Vessel Analyzer (myopia < −8 diopters; hyperopia > 6 diopters; astigmatism > 2 diopters; visual acuity < 0.3).

### Electrical Stimulation

Electrical stimulation at the eye was applied using a neurostimulator (DC-STIMULATOR PLUS, neuroConn GmbH, Ilmenau, Germany) and two non-metallic, conductive rubber electrodes fixed at the subject’s head with elastic straps. This electrode type is used in non-invasive electrical brain stimulation, and the safety of transcranial electrical stimulation methods using these electrodes has been confirmed (Poreisz et al., 2007; Brunoni et al., 2012; Antal et al., 2017). For this study, the electrodes were adapted to a newly developed montage stimulating the central part of the retina rather homogeneously with respect to amplitude and orientation (anterior–posterior) (Hunold et al., 2015). Relevant safety criteria for transcranial direct current stimulation (tDCS) proposed by Nitsche et al. (2003) were considered by our study protocol.

**FIGURE 1:**
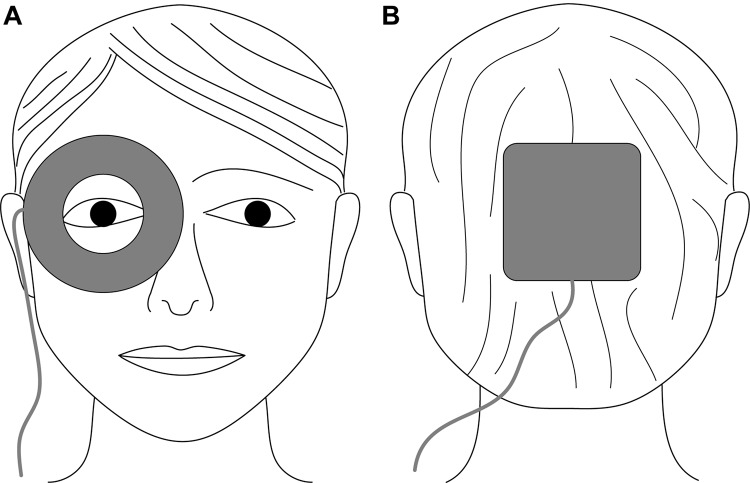
Schematic electrode placement for electrical stimulation of the eye using non-metallic, conductive rubber electrodes. **(A)** Ring-shaped active electrode (inner/outer diameter: 3/7.5 cm) surrounding the examined eye; **(B)** square return electrode (10 cm × 10 cm) placed at the occiput.

For electrical current application, we used flexible rubber electrodes with a different geometry. A ring-shaped active electrode (inner/outer diameter: 3/7.5 cm) was prepared with conductive paste (Ten20, Weaver and Company, Aurora, CO, United States) and placed surrounding the examined eye (Figure 1A). A square return electrode (10 cm × 10 cm) was completely inserted in a saline-soaked (0.9% NaCl solution) sponge and placed at the occiput (Figure 1B). The pulsed electrical stimulation comprised rectangular, monophasic, positive current pulses (pulse width: 50 ms) applied at a frequency of 10 Hz and for a duration of 60 s per stimulation period. Repetition rate and interval were synchronized with the DVA protocol (described below). The pulsed electrical stimulation evokes phosphenes. Therefore, we defined three groups stimulated with different current intensities at approximately 100, 200, or 300% of the phosphene threshold similar to Schatz et al. (2017), resulting in current intensities of 400, 800, and 1200 μA, respectively.

### Dynamic Vessel Analysis

Retinal vessel diameters were determined continuously in the subjects’ eyes using the Retinal Vessel Analyzer (RVA; Imedos Systems UG, Jena, Germany) under different stimulus conditions. The RVA system is used to investigate the flicker light-induced changes of retinal vessel diameters, a procedure known as DVA. The changes are calculated from several provocation and measurement cycles and expressed in the form of maximum vasodilation following the provocation, given in percent of the baseline vessel diameter. The standard RVA device and the DVA procedure are described in detail elsewhere (Seifertl and Vilser, 2002; Garhofer et al., 2010).

**FIGURE 2:**
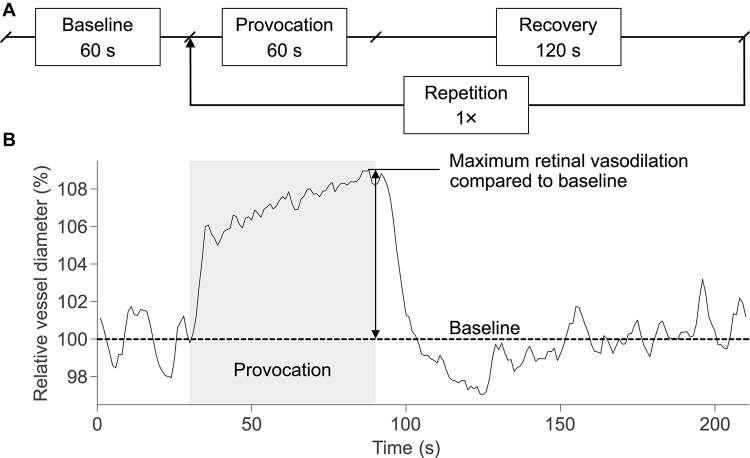
DVA protocol and schematic retinal vessel reaction. **(A)** The DVA protocol in this study consisted of a 60-s baseline measurement, 60 s of provocation, and 120 s of recovery. The provocation and recovery period were repeated once, resulting in a total duration of 420 s for a DVA measurement. **(B)** The retinal vessel reaction was calculated as the mean of both cycles and the maximum retinal vasodilation was determined at the end of the provocation.

The provocation periods in the DVA timing protocol were extended to 60 s for the synchronization with the electrical stimulation duration. Thus, the DVA protocol (Figure 2A) used in this study started with the measurement of baseline vessel diameters for 60 s followed by two cycles of provocation (60 s) and recovery (120 s), resulting in a total duration of 420 s for one DVA measurement. Retinal vessel reactions were calculated as the mean of both cycles and maximum vasodilation was determined for arteries and veins (Figure 2B). During baseline and recovery periods, the eye was illuminated continuously. During provocation periods, electrical and/or flicker light stimulation occurred according to the particular stimulus condition in the experimental procedure. In case of flicker light stimuli, the illumination light was periodically interrupted to create a flicker effect at a frequency of 12.5 Hz (Garhofer et al., 2010).

**FIGURE 3:**
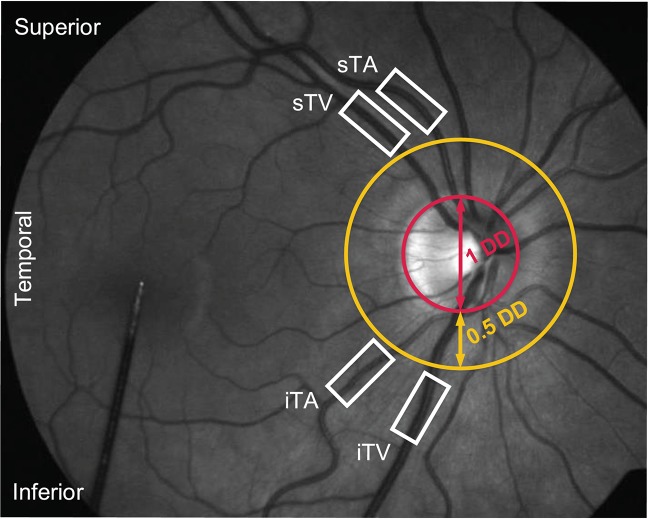
Fundus image of a healthy subject during a DVA measurement. Four primary vessel segments [superior as well as inferior temporal artery (sTA/iTA) and vein (sTV/iTV)] were selected in a distance of at least 0.5 disk diameters (DD) from the optic disk margin.

The pupils of the examined eyes were dilated using tropicamide (Mydiaticum Stulln, Pharma Stulln GmbH, Stulln, Germany) approximately 20 min before starting DVA measurements, since mydriasis is required for DVA. Following the protocol for DVA proposed by Garhofer et al. (2010), up to four primary vessel segments were investigated in each eye, located in a distance of at least 0.5 disk diameters from the optic disk margin (Figure 3): superior temporal artery (sTA); inferior temporal artery (iTA); superior temporal vein (sTV); inferior temporal vein (iTV). The distinction between superior and inferior orientation is based on studies showing differences in retinal blood flow depending on the retinal quadrants (Chung et al., 1999; Garhofer et al., 2012). Individual anatomical structures, for example overlapping or twisted vessels, hindered the selection of all four retinal vessel segments in each subject. Furthermore, pronounced central reflexes on single vessels led to the exclusion from the analyses because diameter measurements could not be performed in these vessels.

### Experimental Procedure

All examinations were conducted in the morning. The subjects were told to ensure a sufficient amount of sleep in the night before and asked to have their individual regular breakfast, which may include the consumption of coffee. Initially, individual thresholds for electrically induced phosphenes and skin sensations were determined using the ascending method of limits. For threshold determination, electrical stimulation from 0 to 1200 μA with 100 μA increments was applied for 5 s. After each increment, the subjects were asked about their perceptions. Phosphene and sensory thresholds were defined as the current intensity at which subjects first reported visual perceptions or skin sensations, respectively.

Subsequently, three DVA measurements with different stimulus conditions during the provocation periods in the DVA protocol were conducted in each subject. The stimulus conditions were as follows: flicker light stimulation (FLS); electrical stimulation (ES); simultaneous electrical and flicker light stimulation (ES+FLS). The current intensity in case of electrical stimulation (ES and ES+FLS) differed between the three groups (400, 800, or 1200 μA). A resting period of approximately 30 min was ensured between the DVA measurements to avoid suppression of retinal vessel reactions in consecutive experiments (Noonan et al., 2013).

### Data Analysis

Retinal vessel reactions were analyzed under the three stimulus conditions (FLS, ES, and ES+FLS) in three groups (400, 800, or 1200 μA). The stimulus condition FLS served as the individual reference measurement as the flicker light-induced vasodilation is well-known in healthy subjects (Kotliar et al., 2004; Nagel and Vilser, 2004; Nagel et al., 2004, 2006; Noonan et al., 2013). To assess the influence of electrical stimulation, the vasodilations after ES and ES+FLS were compared to the vasodilation after FLS.

Measurement results for provocation-induced retinal vasodilation are given as the mean ± standard error of the mean (SEM) of the groups. Statistical analyses were performed using a statistical software (SPSS Statistics 24, IBM Corporation, Armonk, NY, United States). The Shapiro–Wilk test was used to check the vasodilation values of each group and each vessel segment for normal distribution. The normal distribution is given for all measurements, except the iTA measurement in the 1200 μA group. We performed a paired *t*-test to compare the vasodilation values of the different stimulus conditions. Additionally, we applied a robust method based on the comparison of the 20% trimmed mean according to Wilcox (2017) for the iTA measurement in the 1200 μA group because of its unconfirmed normal distribution. All statistical tests were calculated with a significance level of *p* = 0.05. Effect sizes were calculated according to Cohen’s dz (Cohen, 1988; Lakens, 2013).

## Results

### Phosphene and Sensory Thresholds

Electrical stimulation was well-tolerated by all subjects at all applied current intensities. None of the subjects showed side effects during or after the experiments. The phosphene and sensory thresholds (mean ± SEM) were 355.7 ± 16.8 and 353.8 ± 19.1 μA, respectively. The subjects described the skin sensations as weak tingling or pricking sensations perceived under the active electrode. The skin sensations increased with increasing current intensity but were not uncomfortable or painful at any time. The phosphenes appeared in form of diffuse visual flickering sensations that were characterized by the subjects as gentle light–dark changes. The whole visual field of the electrically stimulated eye was covered by the phosphenes and they increased in strength with increased current intensity.

### Retinal Vasodilation

**FIGURE 4:**
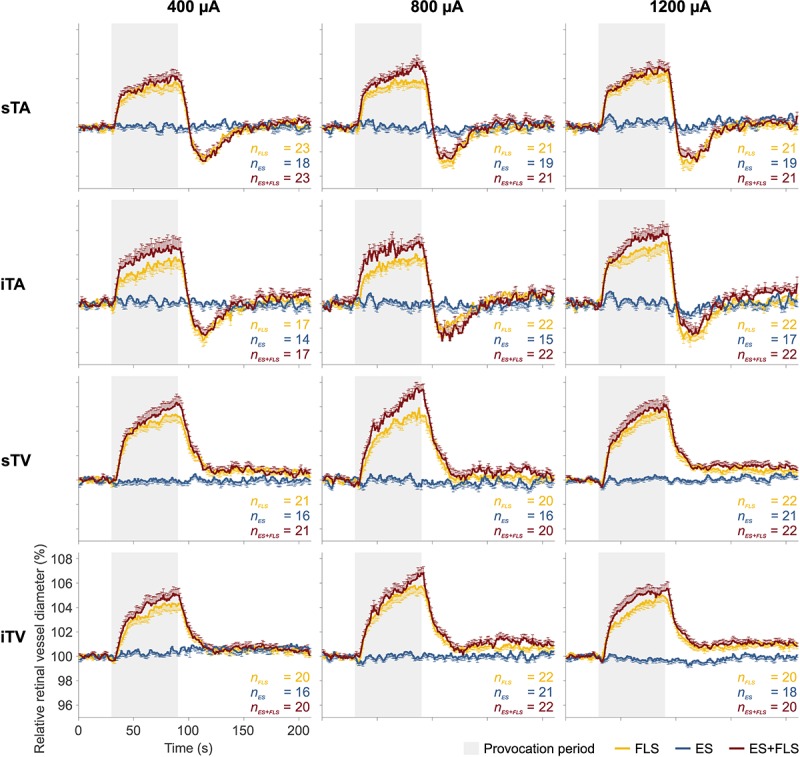
Mean retinal vessel reactions. Each diagram shows the mean retinal vessel reactions under the tested stimulus conditions: flicker light stimulation (FLS); electrical stimulation (ES); electrical and flicker light stimulation (ES+FLS). The left column shows the diagrams of the 400 μA group for the investigated vessel segments (from top to bottom): superior temporal artery (sTA); inferior temporal artery (iTA); superior temporal vein (sTV); inferior temporal vein (iTV). Correspondingly, the middle column shows the diagrams of the 800 μA group and the right column the diagrams of the 1200 μA group. One-sided error bars indicate the standard error of the mean (SEM). Provocation periods are highlighted in gray (30–90 s).

**FIGURE 5:**
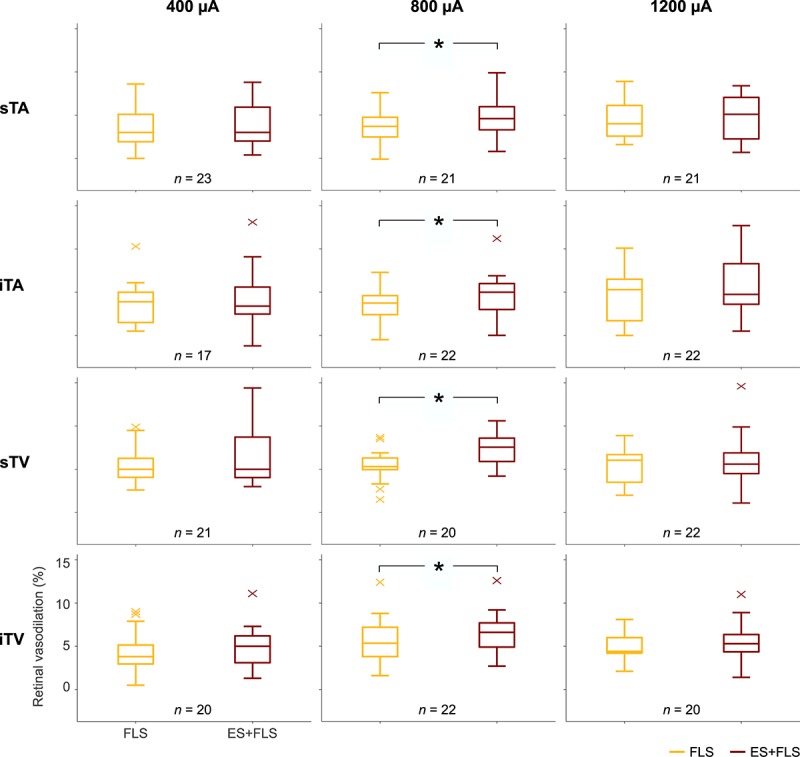
Box-and-whisker plots of retinal vasodilation after provocation with flicker light stimulation (FLS) and electrical and flicker light stimulation (ES+FLS). The left column shows the values of the 400 μA group for the investigated vessel segments (from top to bottom): superior temporal artery (sTA); inferior temporal artery (iTA); superior temporal vein (sTV); inferior temporal vein (iTV). Correspondingly, the middle column shows the diagrams of the 800 μA group and the right column the diagrams of the 1200 μA group. Statistically significant differences are indicated with ^∗^ (paired *t*-test, *p* < 0.05).

The mean retinal vessel reactions under the tested stimulus conditions (FLS, ES, and ES+FLS) are shown in Figure 4 for all groups. The corresponding mean values of retinal vasodilation for stimulus conditions FLS and ES+FLS are given in Table 1 and the graphical representation of the values in box-and-whisker plots is shown in Figure 5. The retinal vasodilation values of the individual subjects are provided in Supplementary Tables S1 (400 μA group), S2 (800 μA group), and S3 (1200 μA group). DVA measurements with FLS induced the expected retinal vessel reactions in all subjects. Generally, the mean vessel diameter increased after the onset of the flickering light and reached its maximum dilation at the end of the provocation period followed by vessel constriction back to baseline diameter. The time courses of arterial vessel reactions show a characteristic undershooting after the end of FLS and before converging to baseline. In case of stimulus condition ES, the provocation induced no changes of the retinal vessel diameters compared to the baseline levels in the examined vessel segments for all groups, regardless of the applied current intensity. Hence, no vasodilation values were calculated and statistically evaluated for the ES experiments. In contrast, DVA measurements using stimulus condition ES+FLS showed retinal vasodilation. The general time courses of the retinal vessel reactions are comparable to the FLS curves but the mean maximum dilation is increased in all groups.

**TABLE 1:** Estimated parameters (mean ± SEM) of retinal vasodilation after provocation with stimulus conditions FLS and ES+FLS statistically compared for each group and vessel segment.

**Current**	**Vessel**	**Mean ± SEM of**		***p*-value for**	**Cohen’s *d*_*z*_**
**intensity**	**segment**	**retinal vasodilation**		** paired *t*-test**	
		**after provocation (%)**			
				
		**FLS**	**ES+FLS**		
400 μA	sTA (*n* = 23)	3.5 ± 0.5	3.9 ± 0.5	0.402	0.18
	iTA (*n* = 17)	3.6 ± 0.6	4.4 ± 0.8	0.152	0.36
	sTV (*n* = 21)	5.3 ± 0.4	6.2 ± 0.6	0.059	0.44
	iTV (*n* = 20)	4.2 ± 0.5	4.9 ± 0.5	0.137	0.35
800 μA	sTA (*n* = 21)	3.7 ± 0.4	4.8 ± 0.5	0.008^∗^	0.64
	iTA (*n* = 22)	3.6 ± 0.4	4.6 ± 0.5	0.023^∗^	0.52
	sTV (*n* = 20)	5.4 ± 0.4	7.2 ± 0.4	< 0.001^∗^	1.29
	iTV (*n* = 22)	5.6 ± 0.5	6.6 ± 0.4	0.004^∗^	0.69
1200 μA	sTA (*n* = 21)	4.4 ± 0.5	4.7 ± 0.5	0.315	0.22
	iTA (*n* = 22)	4.8 ± 0.6	5.6 ± 0.7	0.075	0.40
	sTV (*n* = 22)	5.4 ± 0.4	6.0 ± 0.6	0.214	0.27
	iTV (*n* = 20)	4.8 ± 0.4	5.4 ± 0.5	0.134	0.35

*sTA/iTA, superior/inferior temporal artery; sTV/iTV, superior/inferior temporal vein; FLS, flicker light stimulation; ES+FLS, electrical and flicker light stimulation; d_*z*_, standardized mean difference effect size for repeated measures (within-subjects designs) (Cohen, 1988). ^∗^*p* < 0.05. Interpretation of Cohen’s d_*z*_: small effect (d_*z*_ = 0.2); medium effect (d_*z*_ = 0.5); large effect (d_*z*_ = 0.8) (Cohen, 1988).*

**FIGURE 6:**
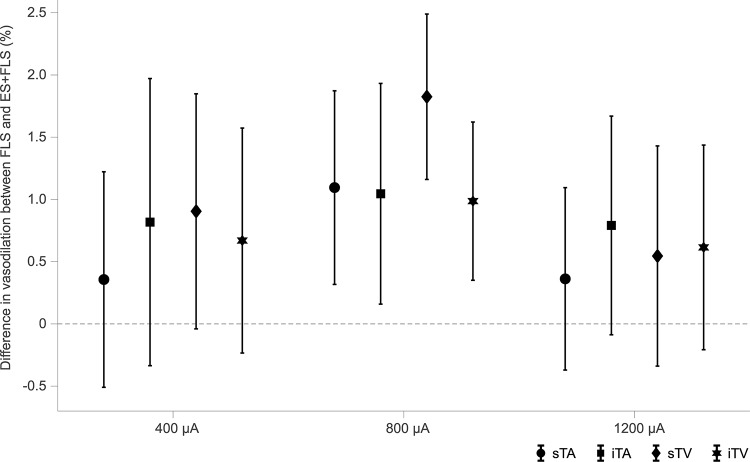
Confidence intervals (confidence level 95%) of the differences between stimulus conditions flicker light stimulation (FLS) and electrical and flicker light stimulation (ES+FLS) for each group and each vessel segment [superior temporal artery (sTA); inferior temporal artery (iTA); superior temporal vein (sTV); inferior temporal vein (iTV)]. Confidence levels that do not include the zero value indicate significant differences.

Statistical analyses (Table 1) display differences in the enhancement of vessel dilation after ES+FLS depending on the applied current intensity of electrical stimulation. The 800 μA group showed significantly increased vasodilation in all four examined vessel segments after ES+FLS compared to FLS. In contrast, the 400 and 1200 μA group showed an upward trend in mean retinal vasodilation for ES+FLS in all vessel segments but no significant differences. Similar to the paired *t*-test, the additionally applied 20% trimmed mean comparison for the iTA measurement in the 1200 μA group yielded a *p*-value of 0.063. The effect sizes of the observed effects (Table 1) can be interpreted based on the Cohen classification (Cohen, 1988). Accordingly, the effects of the 800 μA group were medium to large and the effects of the 400 μA and the 1200 μA group were small. Additionally, we followed the recommendations given by the CONSORT Group (Moher et al., 2010) and analyzed the differences between stimulus conditions FLS and ES+FLS. Figure 6 shows these differences including the confidence intervals (confidence level 95%). Similar to Figure 6, the confidence interval for the 20% trimmed mean of the iTA measurement in the 1200 μA group is [-0.07, 2.21]. Significant differences are indicated by a confidence level that does not include the zero value. This is given for all vessel segments of the 800 μA group and is consistent with the results of the paired *t*-test.

## Discussion

This study investigated the behavior of retinal vessels in response to electrical stimulation using DVA for the first time. We observed a significant enhancement of retinal vasodilation to flickering light in the case of simultaneous electrical stimulation with positive current pulses of 800 μA. In contrast, sole electrical stimulation induced no immediate response of retinal vasculature since the retinal vessel diameter remained unaltered in this stimulus condition compared to baseline.

Transcranial direct current stimulation is known to cause cortical excitability changes by modifying the membrane polarization depending on the polarity of the applied current. In particular, anodal stimulation, which means that positive current is applied at the region of interest, is associated with excitatory effects (Nitsche and Paulus, 2000). In electrical retina stimulation, polarization and depolarization can give rise to phosphenes. Sole electrical stimulation using positive current pulses probably leads to polarization and possibly depolarization (phosphenes) of the retinal cells affected by the electrical stimulation. The retinal vessel diameter does not change under electrical stimulation without any light stimulation. Positive pulsed electrical stimulation seems to modulate the processes, which are responsible for the flicker light-induced vessel dilation. The findings of this work are only valid for retinal vessels in the range of 90–300 μm as this is the current working range of the DVA technique. Retinal vessels < 90 μm might show a different response to ES.

Flickering light presented to the eye is, however, a stimulus that induces modulatory effects regarding neuronal and associated metabolic processes in the healthy human retina. This finding was demonstrated by the flicker light-induced increase of optic nerve head blood flow (Riva et al., 2001) as well as the flicker light-induced increase of retinal vessel diameter (Formaz et al., 1997; Polak et al., 2002). The flickering light causes a short period of heightened cellular metabolism, which increases the need for blood. As a result, the retinal vessels dilate and the blood flow increases, an effect termed functional hyperemia. The assessment of retinal vasodilation in response to flickering light using the DVA system shows a specific retinal vessel reaction in healthy subjects (Kotliar et al., 2004; Nagel and Vilser, 2004; Nagel et al., 2004, 2006; Noonan et al., 2013) that could be seen in our study for stimulus condition FLS as well. In addition to the vasodilation, the arterial reaction comprises a reactive vessel constriction with undershooting under baseline vessel diameter after the end of FLS. This reaction is associated with an overshooting of regulatory processes (Polak et al., 2002; Nagel and Vilser, 2004; Nagel et al., 2004). By combining an additional electrical stimulus with the flickering light (stimulus condition ES+FLS), we observed an enhancement of the retinal vasodilation. This upregulation of the vascular response to flickering light might be associated with an excitatory effect of the electrical stimulation. Thus, the positive current pulses affected retinal neurons and modulated the threshold for activation, resulting in an enhanced retinal vessel reaction to flickering light.

Generally, the modulation of neuronal activity in the retina is associated with variations in local retinal blood flow (Falsini et al., 2002; Riva et al., 2004; Metea and Newman, 2006; Newman, 2015; Noonan et al., 2015), a functional connection known as neurovascular coupling (NVC) (Roy and Sherrington, 1890). In terms of NVC, an activation of retinal neurons results in an increased metabolic demand that is regulated by Müller cells. This major type of retinal glial cells maintains the homeostatic and metabolic support of retinal neurons (Reichenbach and Bringmann, 2013). An adequate stimulus, such as flickering light, provokes a neuronal and metabolic activation that is, among other things, expressed in retinal vessel dilation. In addition to flickering light, voltage-gated ion channels in the membranes of retinal neurons can be influenced by external electrical stimulation; e.g., Pall (2013) described that electromagnetic fields activate voltage-gated calcium channels in cells. Other tissue-level effects of electromagnetic fields are, for example, acceleration of bone fracture healing, changes in brain cognitive functions, neuronal stimulation and neuromuscular stimulation (Adam et al., 1998; Repacholi and Greenebaum, 1999; Funk et al., 2009), stimulation of biosynthesis (Blank and Goodman, 2008), and neuroprotective effects on retinal cells (Pardue and Allen, 2018). Sato et al. (2008a) showed in ocular tissue that electrical stimulation increases the calcium (Ca^2+^) influx through L-type voltage-gated channels and regulates the transcription of neurotrophic factors in cultured Müller cells. An increase in intracellular Ca^2+^ concentration within glial cells is, in turn, supposed to cause the release of neuroactive substances and thus the modulation of neuronal activity (Newman and Zahs, 1997). In addition, it was shown in several studies, which were evaluating the therapeutic effects of electrical stimulation of the eye, that electrical stimulation increases the level of neurotrophic factors that are released from Müller cells, including IGF-1 (Morimoto et al., 2005; Sato et al., 2008b), BDNF (Sato et al., 2008a), and CNTF (Ni et al., 2009). Such impacts of electrical stimulation on retinal neurons might contribute to an upregulation of retinal vessel reactions in response to flickering light.

In the present study, electrical stimulation was applied at three different current intensities (400, 800, and 1200 μA). These values relate to the mean phosphene threshold of the subjects (355.7 ± 16.8 μA) and represent a stimulation near, at double, or at triple phosphene threshold, respectively. Significantly increased vasodilation was observed for stimulus condition ES+FLS in all vessel segments for the 800 μA group. The 400 and 1200 μA group showed an upward trend but no significant effects. These findings correspond to results from studies that evaluated therapeutic impacts of electrical stimulation on ophthalmic parameters in patient groups receiving different intensities of electrical stimulation. Schatz et al. (2011) and Naycheva et al. (2013) treated participants with sham, 66, and 150% of their individual phosphene threshold. Both these studies revealed significant improvements in the visual field (Schatz et al., 2011) and the scotopic a-wave (Naycheva et al., 2013), for the 150% group only. Another study by Schatz et al. (2017) applied electrical stimulation with sham, 150, and 200% of the patients’ individual phosphene threshold. They detected a significantly increased light-adapted single flash b-wave, both in the 150 and 200% group. To summarize, significant changes in the evaluated ophthalmic parameters were only seen in groups where electrical stimulation was applied sufficiently above phosphene threshold, which is consistent with our results for the 400 and 800 μA group. However, we have not seen statistically significant changes in retinal vasodilation to flickering light in the 1200 μA group. This suggests the presence of a non-linear effect. Comparable results were already observed in neuroscientific studies that investigated changes in human motor cortex excitability due to weak tDCS. The dependencies of current intensity, polarity, and duration on stimulation-induced after-effects were analyzed and a non-linear correlation was found (Nitsche and Paulus, 2000; Batsikadze et al., 2013; Jamil et al., 2017). In particular, higher intensity levels of electrical stimulation do not necessarily cause stronger excitability effects.

Further indications for non-linear effects regarding current intensity were found in animal studies that investigated the neuroprotective effects of electrical stimulation in ophthalmic disease models. Morimoto et al. (2010) evaluated the survival of axotomized RGCs in rats after transcorneal electrical stimulation. They observed significantly increased RGC densities after stimulation with 100 and 200 μA but not for lower or higher intensities (50, 300, and 500 μA) compared to sham stimulation.

To evaluate whether electrical stimulation of the human eye induces vasoactive changes in retinal vessel behavior, we performed a mild and well-tolerated stimulation study targeting acute effects only. Hence, the applied electrical stimulation lasted for a short period of 60 s with 50 ms monophasic pulses applied at a 10 Hz repetition frequency (30 s effective stimulation). In contrast, studies targeting after-effects have applied their mostly biphasic stimulation for several minutes, usually 20–40 min.

The generation and control of retinal vascular tone is determined by intrinsic mechanisms (Newman, 2015). Several competing and simultaneously linked autoregulatory mechanisms are involved, including endothelial-mediated regulation (de Wit et al., 2006; Bharadwaj et al., 2013), myogenic mechanisms (Bayliss effect) (Bayliss, 1902; Blum et al., 1999), metabolic mechanisms (Delaey and van de Voorde, 2000; Pournaras et al., 2008), and NVC (Metea and Newman, 2006; Noonan et al., 2015). With our study it is not possible to clarify which of these mechanisms are affected by electrical stimulation.

The present study reveals that flicker light-induced retinal vasodilation is immediately enhanced by electrical stimulation using positive current pulses with an adequate current intensity above the phosphene threshold. The effect of retinal vessel diameter change may partly be associated with the observed beneficial effects of electrical stimulation (Inomata et al., 2007; Gall et al., 2011, 2016; Oono et al., 2011; Sabel et al., 2011; Schatz et al., 2011, 2017; Anastassiou et al., 2013; Naycheva et al., 2013; Chaikin et al., 2015). In future work, the question has to be clarified whether electrical stimulation is also effective in diseases with vascular dysregulation.

## Ethics Statement

This study was approved by the local ethics committee of the Friedrich Schiller University Jena, Germany. All procedures complied with the Declaration of Helsinki and the subjects gave their written informed consent before participating in the study.

## Author Contributions

SF: conceptualization, methodology, data acquisition/curation, data processing/analysis, and manuscript drafting and revision. AH, MK, SK, DL, and EN: conceptualization, methodology, and manuscript revision. JH: project administration/supervision, conceptualization, methodology, and manuscript revision.

## Conflict of Interest

The authors declare that the research was conducted in the absence of any commercial or financial relationships that could be construed as a potential conflict of interest.
