# Corrigendum: Pulsed Electrical Stimulation of the Human Eye Enhances Retinal Vessel Reaction to Flickering Light

**DOI:** 10.3389/fnhum.2020.00167

**Published:** 2020-05-13

**Authors:** Stefanie Freitag, Alexander Hunold, Matthias Klemm, Sascha Klee, Dietmar Link, Edgar Nagel, Jens Haueisen

**Affiliations:** ^1^Institute for Biomedical Engineering and Informatics, Technische Universität Ilmenau, Ilmenau, Germany; ^2^Ophthalmic Private Practice, Rudolstadt, Germany

**Keywords:** pulsed electrical stimulation, transcranial direct current stimulation (tDCS), flicker light stimulation, dynamic vessel analysis, retinal vessel diameter, vasodilation

In the original article, there was an inaccurate statement. The issue arose in discussions with statistical experts, which encouraged us to publish this correction to avoid confusion to the readers of our article. We made the inaccurate statement that the *t*-test is robust against violation of the normal distribution assumption; however, this only applies under certain conditions. To assure our choice of the paired *t*-test for the data with unconfirmed normal distribution (iTA measurement in the 1200 μA group) we used an additional robust method for this data. The results of the robust method that is based on the comparison of the 20% trimmed mean according to Wilcox (2017) confirm the results of the paired *t*-test.

A correction has been made to the **Materials and Methods** section, subsection **Data Analysis**, paragraph 2:

“Measurement results for provocation-induced retinal vasodilation are given as the mean ± standard error of the mean (SEM) of the groups. Statistical analyses were performed using a statistical software (SPSS Statistics 24, IBM Corporation, Armonk, NY, United States). The Shapiro-Wilk test was used to check the vasodilation values of each group and each vessel segment for normal distribution. The normal distribution is given for all measurements, except the iTA measurement in the 1200 μA group. We performed a paired *t*-test to compare the vasodilation values of the different stimulus conditions. Additionally, we applied a robust method based on the comparison of the 20% trimmed mean according to Wilcox (2017) for the iTA measurement in the 1200 μA group because of its unconfirmed normal distribution. All statistical tests were calculated with a significance level of *p* = 0.05. Effect sizes were calculated according to Cohen's *d*_*z*_ (Cohen, 1988; Lakens, 2013).”

A further correction has been made to the **Results** section, subsection **Retinal Vasodilation**, paragraph 2:

“Statistical analyses **(Table 1)** display differences in the enhancement of vessel dilation after ES+FLS depending on the applied current intensity of electrical stimulation. The 800 μA group showed significantly increased vasodilation in all four examined vessel segments after ES+FLS compared to FLS. In contrast, the 400 μA and the 1200 μA group showed an upward trend in mean retinal vasodilation for ES+FLS in all vessel segments but no significant differences. Similar to the paired *t*-test, the additionally applied 20% trimmed mean comparison for the iTA measurement in the 1200 μA group yielded a *p*-value of 0.063. The effect sizes of the observed effects **(Table 1)** can be interpreted based on the Cohen classification (Cohen, 1988). Accordingly, the effects of the 800 μA group were medium to large and the effects of the 400 μA and the 1200 μA group were small. Additionally, we followed the recommendations given by the CONSORT Group (Moher et al., 2010) and analyzed the differences between stimulus conditions FLS and ES+FLS. **Figure 6** shows these differences including the confidence intervals (confidence level 95%). Similar to **Figure 6**, the confidence interval for the 20% trimmed mean of the iTA measurement in the 1200 μA group is [−0.07, 2.21]. Significant differences are indicated by a confidence level that does not include the zero value. This is given for all vessel segments of the 800 μA group and is consistent with the results of the paired *t*-test.”

The authors apologize for this error and state that this does not change the scientific conclusions of the article in any way. The original article has been updated.
