# Supplementary material for: Pulsed Electrical Stimulation of the Human Eye Enhances Retinal Vessel Reaction to Flickering Light
Source: Front Hum Neurosci. 2019 Oct 22;13:371. doi: 10.3389/fnhum.2019.00371 (PMC6817672; doi:10.3389/fnhum.2019.00371)
Supplement: Supplementary file 1 [file Table_1.pdf]

Supplementary Table S1. Retinal vasodilation values of the individual subjects of the 400 µA group after provocation with stimulus conditions FLS and ES+FLS.

**Subject**	**FLS**				**ES+FLS**			
	**sTA**	**iTA**	**sTV**	**iTV**	**sTA**	**iTA**	**sTV**	**iTV**
s_01	0.0	2.2	4.7	-	3.0	2.7	8.7	-
s_02	4.8	-	-	-	4.5	-	-	-
s_03	3.0	6.1	-	7.9	1.5	8.0	-	6.2
s_04	2.3	0.8	6.2	5.1	2.9	3.0	4.6	7.0
s_05	0.5	3.9	7.5	2.7	1.4	3.2	9.4	3.3
s_06	4.1	0.9	6.7	-	3.6	4.8	9.1	-
s_07	8.6	10.3	5.3	5.2	8.8	13.1	5.5	5.8
s_08	7.8	-	-	-	7.3	-	-	-
s_09	-	-	4.2	0.5	-	-	4.2	3.8
s_10	1.0	-	5.0	3.3	2.3	-	9.2	5.5
s_11	4.3	5.5	9.5	8.7	2.4	3.8	6.9	6.2
s_12	3.0	-	4.0	3.2	0.4	-	6.0	4.6
s_13	3.5	5.3	-	4.0	6.2	8.4	-	5.4
s_14	2.3	-	-	5.2	2.6	-	-	4.0
s_15	-	2.2	6.5	-	-	4.4	8.8	-
s_16	3.4	4.6	3.0	3.9	6.0	9.1	5.0	6.2
s_17	1.4	2.0	2.7	3.3	5.7	4.4	4.5	7.3
s_18	5.6	4.9	5.7	2.2	4.1	3.4	3.7	2.9
s_19	5.4	1.7	5.0	5.0	0.8	1.2	4.1	2.2
s_20	5.2	4.4	9.9	9.0	7.1	3.2	14.4	11.1
s_21	3.0	4.8	4.2	4.0	4.6	1.8	4.9	6.0
s_22	-	-	4.1	-	-	-	3.0	-
s_23	6.4	0.8	5.2	3.7	8.3	1.5	7.0	4.6
s_24	1.1	-	2.6	2.1	1.1	-	3.9	1.9
s_25	2.0	-	5.0	2.1	1.9	-	3.4	2.9
s_26	1.9	0.5	3.6	3.7	2.3	-1.2	3.3	1.3

sTA/iTA, superior/inferior temporal artery; sTV/iTV, superior/inferior temporal vein; FLS, flicker light stimulation; ES+FLS, electrical and flicker light stimulation
