# Supplementary material for: Pulsed Electrical Stimulation of the Human Eye Enhances Retinal Vessel Reaction to Flickering Light
Source: Front Hum Neurosci. 2019 Oct 22;13:371. doi: 10.3389/fnhum.2019.00371 (PMC6817672; doi:10.3389/fnhum.2019.00371)
Supplement: Supplementary file 2 [file Table_2.pdf]

Supplementary Table S2. Retinal vasodilation values of the individual subjects of the 800 µA group after provocation with stimulus conditions FLS and ES+FLS.

Subject	FLS				ES+FLS			
	sTA	iTA	sTV	iTV	sTA	iTA	sTV	iTV
s_27	4.0	-	6.5	3.5	4.7	-	7.6	4.3
s_28	2.6	3.2	3.3	2.5	3.8	5.0	4.2	2.7
s_29	2.8	-0.5	5.9	4.3	3.3	3.0	9.0	8.2
s_30	4.7	5.9	6.3	4.3	9.9	6.8	7.7	4.7
s_31	3.8	7.3	8.5	1.6	4.0	4.7	8.1	4.8
s_32	1.1	2.4	5.2	3.3	1.8	4.2	5.1	4.9
s_33	7.6	3.7	2.7	5.6	8.5	6.2	4.2	6.7
s_34	-	4.4	2.7	5.6	-	11.2	6.1	5.4
s_35	4.1	3.7	-	12.4	5.2	5.4	-	12.6
s_36	3.7	4.4	5.3	8.8	4.0	4.5	6.2	6.7
s_37	-	4.4	-	-	-	5.0	-	-
s_38	5.8	5.1	6.0	7.1	6.7	5.9	8.0	8.2
s_39	2.6	-	-	-	5.8	-	-	-
s_40	-	5.1	6.0	7.2	-	6.9	8.3	7.5
s_41	-	4.6	5.0	6.3	-	5.3	5.9	6.2
s_42	4.9	-	5.3	3.8	4.6	-	10.2	6.5
s_43	-0.1	-	-	5.1	0.8	-	-	7.9
s_44	2.1	1.9	1.5	4.3	6.2	0.0	6.0	7.2
s_45	6.2	5.2	8.7	8.2	4.7	6.0	10.6	9.2
s_46	2.9	0.5	-	-	2.1	1.0	-	-
s_47	4.4	3.7	6.9	4.2	3.6	6.1	8.9	5.4
s_48	6.5	4.6	6.3	7.2	8.2	4.0	9.0	7.7
s_49	3.7	1.4	5.1	3.5	3.3	2.2	5.1	4.8
s_50	2.1	2.7	5.0	-	5.9	5.5	7.5	-
s_51	1.9	-	-	-	3.3	-	-	-
s_52	-	1.6	4.9	6.2	-	0.7	5.9	5.4
s_53	-	3.8	-	7.9	-	2.5	-	7.6

sTA/iTA, superior/inferior temporal artery; sTV/iTV, superior/inferior temporal vein; FLS, flicker light stimulation; ES+FLS, electrical and flicker light stimulation
