# Supplementary material for: Pulsed Electrical Stimulation of the Human Eye Enhances Retinal Vessel Reaction to Flickering Light
Source: Front Hum Neurosci. 2019 Oct 22;13:371. doi: 10.3389/fnhum.2019.00371 (PMC6817672; doi:10.3389/fnhum.2019.00371)
Supplement: Supplementary file 3 [file Table_3.pdf]

Supplementary Table S3. Retinal vasodilation values of the individual subjects of the 1200 µA group after provocation with stimulus conditions FLS and ES+FLS.

Subject	FLS				ES+FLS			
	sTA	iTA	sTV	iTV	sTA	iTA	sTV	iTV
s_54	8.2	7.2	6.1	5.0	8.4	9.5	5.6	5.7
s_55	3.2	8.3	-	5.9	7.0	10.4	-	8.1
s_56	3.2	0.9	8.9	8.1	6.2	4.1	14.6	8.9
s_57	5.2	9.5	6.9	6.2	5.1	8.4	8.1	11.0
s_58	6.2	6.3	6.7	-	7.2	8.3	6.9	-
s_59	8.9	10.1	6.5	4.2	7.6	12.7	5.6	5.3
s_60	1.6	5.4	6.1	4.3	1.8	7.0	5.1	4.8
s_61	-	6.1	8.3	-	-	6.1	6.4	-
s_62	-	6.5	2.2	6.1	-	2.2	1.1	2.4
s_63	-	5.5	2.8	-	-	4.1	1.6	-
s_64	-	0.0	2.5	-	-	1.2	4.7	-
s_65	2.6	1.5	-	-	4.1	4.1	-	-
s_66	2.7	-	3.5	3.5	0.7	-	6.8	6.3
s_67	1.6	3.5	5.9	4.2	3.5	5.4	8.7	4.7
s_68	-	-	-	4.4	-	-	-	6.8
s_69	5.5	2.4	-	7.3	2.1	0.5	-	6.4
s_70	4.8	5.6	6.4	7.0	6.3	7.3	3.6	5.3
s_71	1.8	4.3	5.7	4.9	1.3	3.1	5.6	5.1
s_72	6.1	1.6	6.1	4.2	7.2	3.7	6.4	3.5
s_73	4.3	5.2	2.0	2.1	4.2	3.7	2.9	1.4
s_74	6.6	4.9	4.0	2.1	6.7	8.0	4.5	3.0
s_75	7.1	8.8	7.0	4.4	7.2	9.9	8.0	5.3
s_76	4.0	-	-	-	5.2	-	-	-
s_77	2.5	1.1	3.5	3.5	2.3	0.5	6.7	4.0
s_78	-	-	8.2	-	-	-	9.9	-
s_79	2.4	-	4.5	4.5	3.1	-	3.8	5.1
s_80	3.0	1.7	6.0	4.4	1.9	3.6	5.2	5.5

sTA/iTA, superior/inferior temporal artery; sTV/iTV, superior/inferior temporal vein; FLS, flicker light stimulation; ES+FLS, electrical and flicker light stimulation
